# CRISPR/Cas9‐mediated gene knockout for DNA methyltransferase Dnmt3a in CHO cells displays enhanced transgenic expression and long‐term stability

**DOI:** 10.1111/jcmm.13687

**Published:** 2018-05-30

**Authors:** Yan‐Long Jia, Xiao Guo, Jiang‐Tao Lu, Xiao‐Yin Wang, Le‐Le Qiu, Tian‐Yun Wang

**Affiliations:** ^1^ College of Pharmacy Xinxiang Medical University Xinxiang Henan China; ^2^ International Joint Research Laboratory for Recombiant Pharmaceutical Protein Expression System of Henan Xinxiang Medical University Xinxiang Henan China; ^3^ School of Basic Medicine Xinxiang Medical University Xinxiang Henan China

**Keywords:** Chinese hamster ovary cell, DNA methylation, Dnmt3a, gene knockout, transgene expression

## Abstract

CHO cells are the preferred host for the production of complex pharmaceutical proteins in the biopharmaceutical industry, and genome engineering of CHO cells would benefit product yield and stability. Here, we demonstrated the efficacy of a Dnmt3a‐deficient CHO cell line created by CRISPR/Cas9 genome editing technology through gene disruptions in Dnmt3a, which encode the proteins involved in DNA methyltransferases. The transgenes, which were driven by the 2 commonly used CMV and EF1α promoters, were evaluated for their expression level and stability. The methylation levels of CpG sites in the promoter regions and the global DNA were compared in the transfected cells. The Dnmt3a‐deficent CHO cell line based on Dnmt3a KO displayed an enhanced long‐term stability of transgene expression under the control of the CMV promoter in transfected cells in over 60 passages. Under the CMV promoter, the Dnmt3a‐deficent cell line with a high transgene expression displayed a low methylation rate in the promoter region and global DNA. Under the EF1α promoter, the Dnmt3a‐deficient and normal cell lines with low transgene expression exhibited high DNA methylation rates. These findings provide insight into cell line modification and design for improved recombinant protein production in CHO and other mammalian cells.

## 1 INTRODUCTION

Mammalian cells, particularly Chinese hamster ovary (CHO) cell lines, are typically used for the production of recombinant therapeutic proteins because these cells offer advantages in assembling and folding complex polypeptides and in facilitating post‐translational modifications.^1^, ^2^ The loss of productivity is commonly reported in recombinant CHO cells and is a main concern during long‐term cultivation.^3^, ^4^, ^5^ Expression stability has become a major prerequisite for the large‐scale production of protein therapeutics in mammalian cells. Therefore, recombinant protein production is still challenged by the lack of cell lines that can achieve both high production and stability.^6^, ^7^

The actual mechanisms underlying subclonal heterogeneous expression patterns and product instability remain unclear. A possible reason for the decrease in transgene expression is the gradual loss of gene copies, which result in decreased transcripts 8, 9 and transcriptional silencing.^4^ These problems could be overcome by including epigenetic regulatory DNA elements, such as matrix attachment regions,^10^, ^11^ ubiquitous chromatin opening elements 12, 13, 14 and insulators.^15^ Transgene silencing is also associated with the methylated cytosine on the CpG sites of promoters in recombinant protein‐producing CHO cells.^16^ Yang et al^17^ found an increase in the methylation of the human cytomegalovirus major immediate‐early (hCMV‐MIE) promoter, which controls the monoclonal antibody expression in unstable cell lines with low productivity. Moreover, they discovered that the lost productivity of specific mAb can be partially restored by treating these cells with 5‐Aza‐2‐deoxycytidine (which is a DNA methylation inhibitor). These findings suggested that epigenetic factors, such as DNA methylation, possibly contribute to the loss of productivity and play important roles in regulating gene expression.^18^ Therefore, elucidating the effects of DNA methylation and its mechanism on transgene expression would benefit the development of recombinant CHO cell lines that produce both high and stable production.

Previous studies on the effect of DNA methylation on the transgene expression in CHO cells have mainly focused on promoter modification, such as mutating the cytosine within promoters,^19^ using CpG‐free 20 or synthetic promoters 21 and inserting core CpG island elements into promoters.^22^ Nonetheless, although a promoter without CpG dinucleotides could mitigate early gene silencing, it still cannot improve the long‐term expression stability in transfected CHO cells.^20^ DNA methylation is catalysed by DNA methyltransferases, including those which establish methylation (Dnmt3a and Dnmt3b) 23 and maintain methylation (Dnmt1).^18^ In particular, Dnmt3a and Dnmt3b mediate epigenetic silencing through histone modification and subsequently DNA methylation.^24^ As inhibitors, quinolone analogues are potent against human Dnmt3a, which can induce the re‐expression of a reporter gene controlled by a methylated CMV promoter in leukaemia KG‐1 cells.^25^ Thus, we proposed that modifying the CHO cell line through the gene knockout of DNA methyltransferase would improve the expression stability of permanently transfected CHO cells during long‐term cultivation. To validate this hypothesis, we knocked out the Dnmt3a gene through CRISPR/Cas9 genome editing technology to establish a Dnmt3a‐deficient CHO cell line and investigated the transgene expression level and stability in the deficient cells.

## 2 MATERIALS AND METHODS

### 2.1 sgRNA target design for Dnmt3a KO and plasmid construction

A DNA fragment 3a3 + 4 of Dnmt3a (NW_003613640.1) containing exon1 was amplified by polymerase chain reaction (PCR) from the genomic DNA of CHO‐K1 cells using primers (Table 1). Two pairs of sgRNAs used for targeting the exon1 of Dnmt3a (Table 1) were designed and synthesized (TaKaRa, Dalian, China). If the first nucleotide was not G, we added an additional G nucleotide at the 5′ end, because the U6 promoter prefers a G nucleotide for transcriptional initiation. The sense and antisense single‐stranded oligos (100 mmol/L) were annealed in NEBuffer4 (New England Biolabs, Ipswich, MA, USA) by incubating the oligos mix at 95°C for 5 minutes. The annealed sgRNA oligos were cloned into pX330 vectors (Addgene, #42230) by conducting Bbs I digestion and ligation on the hybridized oligos, and the resulting CRISPR vectors were confirmed by sequencing and were referred to as pX330‐3a1 and pX330‐3a2, respectively. The plasmid was purified for cell transfection using an EndoFree Maxi Plasmid Kit (Tiangen, Beijing, China).

**Table 1:** Primers used for PCR amplification and gene knockout

Gene	Primers	Sequences (5′‐3′)	Size, bp
Dnmt3a 3 + 4	D3a‐Ex1seq‐L	GACCACAAGAATTCCGGCTC	493
	D3a‐Ex1seq‐R	CGTGTGTGAATCTGTGTGGG	
sgRNA1	D3a‐Ex1‐98rev	ATCATCCTCCCGCTCCAAAGTGG	
sgRNA2	D3a‐Ex1‐308fw	TTTGAGGGGTCATCCTTGCAGGG	
CMV promoter	CMV_8F_10F	aggaagagagTGATTTTATGGGATTTTTTTATTTGG	284
	CMV_8F_T7R	cagtaatacgactcactatagggagaaggctTTCTCTAATTAACCAAAAAACTCTACTT	
EF‐1α promoter	EF‐1α_2F_10F	aggaagagagTTATTATTGAGGTGGAGAAGAGTATG	459
	EF‐1α_2F_T7R	cagtaatacgactcactatagggagaaggctCAAACCAAACCTCAACTCAAACA	
Dnmt3a for qPCR	F	CGATGAACCGGAGTACGAGG	179
	R	CCACTGAGAACTTGCCGTCT	
GAPDH for qPCR	F	CCGCATCCCTGAGACAAGAT	175
	R	TGCCGTGGGTGGAATCATAC	

### 2.2 Selection and identification of monoclonal Dnmt3a KO CHO cells

CHO‐K1 cells acquired from Life Technologies (Carlsbad, CA, USA) were grown in Dulbecco's modified Eagle's medium + F12 (Gibco, Carlsbad, CA), supplemented with 10% foetal bovine serum (Gibco, Grand Island, NY, USA), 1% penicillin and streptomycin (Beyotime, Shanghai, China). The cells were incubated in a humidified incubator at 37°C under 5% CO_2_. The cells were passaged every 3 days by diluting the cells to 2 × 10^5^ cells/mL. For the Dnmt3a KO experiment, the CHO‐K1 cells were plated in a 24‐well plate at 24 hours prior to transfection with 80%‐90% confluency. Approximately 2.0 × 10^5^ of the cells in each well was cotransfected with plasmids pX330‐3a1 and pX330‐3a2 (500 ng each) using the Lipofectamine^®^ 3000 Transfection Reagent (Invitrogen, Carlsbad, CA, USA) in accordance with the manufacturer's protocol. After being transfected for 8 hours, the cells in the plate were recovered for 48 hours in fresh medium and the single clones were screened. The transfected cells were subjected to gradient and limiting dilution to select the single clones of Dnmt3a KO cells. These single putative clones were identified through PCR amplification using the sequencing primers (Table 1). The expected PCR products were cloned into the pMD18‐T vector (TaKaRa) and sequenced for further confirmation.

Quantitative detection in vitro was performed using DNMT3A ELISA kit (RenJie, Shanghai, China) to determine the concentrations of Dnmt3A in the Dnmt3a KO CHO‐K1 cells. After the stop solution was added, the blue colour changed to yellow, and the intensity was measured using a spectrophotometer at 450 nm. The calibration standards were also assayed. The concentrations of Dnmt3A in the cellular samples were determined by comparing the OD of the samples to the standard curve.

### 2.3 Dnmt 3a mRNA analysis using quantitative real‐time PCR (qPCR)

qPCR was conducted to determine the expression levels of Dnmt3a mRNA in the Dnmt3a‐deficient and normal control CHO‐K1 cells. Briefly, total RNAs were extracted from the cells using RNAiso Plus (TaKaRa) and reverse transcribed with the PrimeScript^™^ II 1st Strand cDNA Synthesis Kit (TaKaRa) according to the manufacturer's instructions. qRT‐PCR was performed with SYBR Green using a SYBR Premix EX Taq^™^ (TaKaRa) in a final reaction volume of 10 μL: 4 μL of template cDNA (0.05 μg/μL), 5 μL of SYBR Premix Ex Taq (2×), 0.2 μL (10 μmol/L) of forward primer, 0.2 μL (10 μmol/L) of reverse primer and 0.6 μL of deionized water. The qPCR was carried out on a PikoReal^™^ 96 Real‐Time PCR System (ThermoFisher Scientific, USA) equipped with PikoReal Software 2.2 (ThermoFisher Scientific). The amplification conditions were as follows: 95°C for 3 minutes, followed by 40 cycles of 95°C for 15 seconds, 60°C for 20 seconds and 72°C for 10 seconds, and a final extension at 72°C for 5 minutes. Finally, relative expression levels of Dnmt3a mRNA were calculated using the 2^ΔΔCt^ method, with normalization to GAPDH mRNA levels as an internal control. The primers used in this study are shown in Table 1. All the reactions were repeated 3 times.

### 2.4 Protein extraction and Western blot analysis

Western blot was used to identify the protein levels of Dnmt3a in the Dnmt3a‐deficient and normal control CHO‐K1 cells. Briefly, the pellets of the cells were lysed on ice using the extraction buffer (150 nM NaCl, 50 mmol/L Tris HCl pH 7.5, 0.5% SDS, 30 mmol/L PPi, NaF 0.5 mol/L, 100 μmol/L Na_3_VO_4_ 20 mmol/L). A protease inhibitors cocktail (Sigma) was added before extraction. Cell lysates of the samples were separated by 10% SDS‐PAGE gel (Mini‐PROTEAN TGX gels) and transferred to nitrocellulose membrane (Amersham Protran 0.2 μm; GE Healthcare Life Science). Western blot was performed using primary antibodies anti‐DNMT3a (anti‐Dnmt3a ab4897; Abcam) and anti‐GAPDH (Santa Cruz Bitotechnology, Inc.) at dilutions 1:1000. Secondary antibodies conjugated with HRP were used at a dilution of 1:2000, and the reaction was detected using Amersham ECL Prime Western Blotting Detection Reagent by GE Healthcare Life Sciences, according to the manufacturer's instructions.

### 2.5 Construction of the expression vectors and transient transfection

The pWTY‐CMV and pWTY‐EF1α vectors, which contained either the CMV (589 bp) or the EF1α (1335 bp) promoters, were constructed by cloning eGFP as a report gene from pEGFP‐C1 (Clontech, USA) into the pIRES‐neo vector (Clontech).^26^ The sequences of the elements were synthesized and sequenced by General Biosystems (Chuzhou, Anhui, China). Transfection was performed with freshly prepared plasmid DNA. Briefly, the Dnmt3a‐deficient and normal control CHO‐K1 cell lines were counted, adjusted to 2 × 10^5^ cells/mL and then plated into 24‐well plates at 1 mL/well. When 80%‐90% confluence was achieved, the cells in each well were transfected with equimolar amounts of pWTY‐CMV and pWTY‐EF1α using Lipofectamine^®^ 3000 Transfection Reagent (Invitrogen) in accordance with the manufacturer's protocol. At 48‐hour post‐transfection, the transfection efficiency and the transient expression were analysed by assessing the fluorescence intensity of the transfected cells through fluorescence microscopy (Nikon ECLIPSE Ti, Nikon, Japan).

### 2.6 Analysis of cell characteristics

Cell viability was measured using Cell Counting Kit‐8 (Dojindo, Kamimashiki‐gun, Kumamoto, Japan) in accordance with the manufacturer's instructions. Briefly, the CHO‐K1 cells, the deficient cells and the cells stably transfected with CMV or EF1α were seeded in four 96‐well plates at a density of 2 × 10^3^ cells/well. One plate was removed at the same time every day after the cells adhered to the wells. Absorbance was measured with a microplate reader at 450 nm. All experiments were performed in triplicate. Cell cycle and the apoptosis rate were analysed through flow cytometric analysis. Cells were seeded into a 35‐mm dish at a density of 1 × 10^5^ cells/dish. After 72 hours, the harvested cells were washed with cold PBS thrice and fixed in 70% ethyl alcohol at 4°C overnight. For apoptosis analysis, the cells were stained using Annexin V‐FITC Apoptosis Detection Kit (Beyotime Biotechnology, Shanghai, China) in accordance with the manufacturer's instructions. Flow cytometric analysis was performed using the Guava EasyCyte 6‐2L system (Merckmillipore, USA), and data were analysed by Guava InCyte (GuavaSoft^™^ 3.2).

The cell doubling time (*t*_D_) was determined to evaluate the growth characteristics of the normal CHO‐K1 cells, the Dnmt3a‐deficient cells and the cells stably transfected with CMV or EF1α. Briefly, the cells were seeded into 6‐well plates at 1 × 10^5^ cells/mL and allowed to grow for 2, 4, 6, 10, 24 and 48 hours at 37°C under 5% CO_2_ prior to cell counting using a haemocytometer measurement tool and trypan blue staining assay. The *t*_D_ value was determined by the formula *t*_D_ = (*t*_2_−*t*_1_) × (log(2)/log(*C*_2_/*C*_1_)), where *C*_2_ and *C*_1_ are the cell concentrations at times *t*_2_ and *t*_1_, respectively, under a constant growth rate.

### 2.7 Long‐term stability analysis for stably transfected cells

Stably transfected cell polycolonies were selected by adding Geneticin (G418, Invitrogen) to the medium at a final concentration of 800 μg/mL for 14 days. Cells stably transfected with CMV or EF1α were split into 2 and further passaged in either the presence (400 mg/mL, G418^+^) or the absence (G418^−^) of G418 for 60 generations. The mean fluorescent intensity (MFI) values of each cell line at 0, 10, 20, 30, 40, 50 and 60 passages were measured with a FACS Calibur cytometer (Becton Dickinson, Franklin Lakes, NJ, USA). Detailed protocols for stability testing were based on a previous report.^27^

### 2.8 Global DNA methylation assessment

Genomic DNA was extracted from stably transfected cells using the TIANamp Genomic DNA Kit (Tiangen Biotech, Beijing, China) and the Tissue and Cell Genomic DNA Purification Kit (GeneMark). Global DNA methylation levels were determined using MethylFlash^™^ Global DNA Methylation (5‐methylcytosine, 5‐mC) ELISA Easy Kit (EPIGENTEK, Farmingdale, NY) in accordance with the manufacturer's instruction. Briefly, the methylated DNA was detected by using capture and detection antibodies to (5‐mC) and then quantified calorimetrically using SpectraMax^®^ i3 Platform (Molecular Devices, Sunnyvale, CA, USA) to read the absorbance at 450 nm. The amount of methylated DNA was proportional to the measured OD intensity. A standard curve was used to quantify the absolute amount of methylated DNA (the percentage of 5‐mC) in the total genomic DNA. Each sample was assessed in duplicate.

### 2.9 Promoter methylation patterns by MALDI‐TOF mass array

Quantitative methylation changes in CMV and EF1α in the transfected cells were compared using high‐throughput mass spectrometry on the matrix‐assisted laser desorption/ionization time‐of‐flight (MALDI‐TOF) mass array. Briefly, genomic DNA was extracted from CHO cell polycolonies stably transfected with the vectors containing CMV or EF1α at the start and at 50 passages of stability testing. Isolated genomic DNA was treated with bisulphite using the EZ DNA Methylation Kit (Zymo Research, USA). We designed primers for CMV and EF1α to cover the regions with the main CpG sites (Table 1). For PCR amplification, a T7‐promoter tag was added to the reverse primer, and a 10‐mer tag sequence was added to the forward primer to balance PCR primer length. The following PCR programs were used to amplify the bisulphite‐treated genomic DNA with HotStarTaq DNA Polymerase (Qiagen): one cycle at 95°C for 4 minutes; 48 cycles of 95°C for 20 seconds, 56°C for 30 seconds and 72°C for 60 seconds; one cycle at 72°C for 3 minutes; hold at 4°C.

T Cleavage assay was performed using the “T” Cleavage MassCLEAVE^™^ Reagent Kit (SEQUENOM, San Diego, CA, USA). Unincorporated dinucleotide triphosphates were removed by inducing shrimp alkaline phosphatase (SEQUENOM) treatment. The T Cleavage Transcription/RNase Cocktail (5 μL) contained 3.15 μL of RNase Free‐ddH_2_O, 0.89 μL of 5× T7 polymerase buffer, 0.24 μL of T Cleavage mix, 0.22 μL of 100 mmol/L DTT, 0.44 μL of T7 RNA and DNA polymerase (22 units/reaction) and 0.06 μL of RNase A. Afterward, 2 μL of the PCR product (as the template) and the aforementioned 5 μL of MassCLEAVE mix were added to one well of the 384‐well plate for the transcription reaction at 37°C for 3 hours. The samples were diluted with H_2_O to a final volume of 27 μL. The phosphate backbone was conditioned by adding 6 mg of Clean Resin (SEQUENOM) prior to MALDI‐TOF MS analysis. Exactly 22 μL of the RNase‐A‐treated product was robotically dispensed onto silicon matrix preloaded chips (G384 + 10 Spectrochip^™^; SEQUENOM). Mass spectra were obtained using MassARRAY Compact MALDI‐TOF (SEQUENOM), and methylation ratios of the spectra were generated using EpiTYPER software v1.0 (SEQUENOM).

### 2.10 Statistical analysis

The data were expressed as means ± standard deviation. All statistical analyses were performed on the SPSS 18.0 (SPSS, Inc., Chicago, IL, USA). One‐way ANOVA was used to compare multiple groups. Paired‐sample Student's *t* test was used for statistical analysis when only 2 groups were tested. *P* < .05 was considered statistically significant. All experiments were performed at least thrice, and all samples were tested in triplicate.

## 3 RESULTS

### 3.1 Dnmt3a KO by CRISPR/Cas9 genome editing

**Figure 1:**
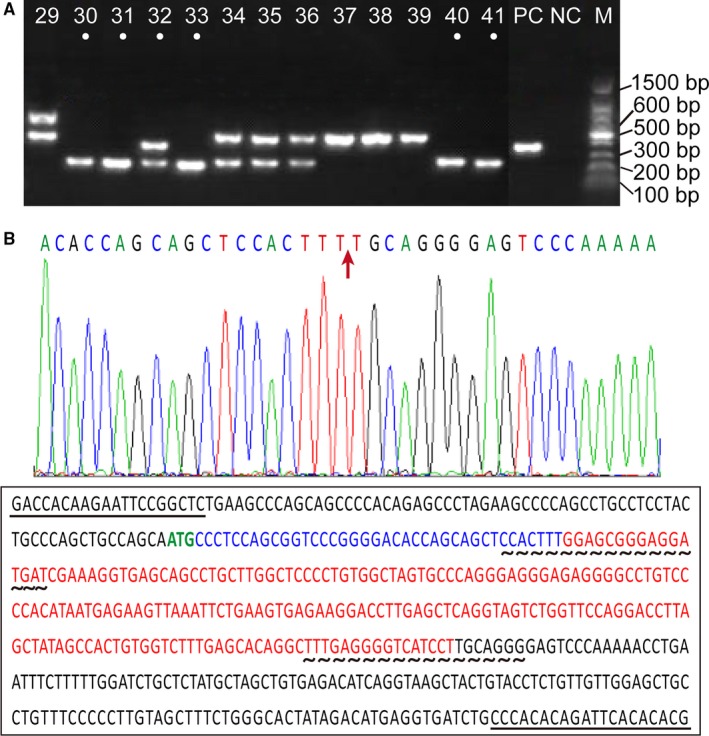
Identification of Dnmt3a KO using the CRISPR/Cas9 system in CHO‐K1 cells. A, PCR amplification for Dnmt3a gene in the monoclones of CHO‐K1 cells.. Six monoclones (3a‐30, 31, 32, 33, 40 and 41) harbouring indel mutations, which cause PCR product length polymorphisms, were selected as Dnmt3a‐deficient mutants. B, Sequencing analysis of Dnmt3a KO in the monoclones 3a‐30 and 40. Sequencing results show that frame shift mutation (red arrow) occurred in the target region of the Dnmt3a gene (the bases in red). Sequencing primers are underlined. sgRNAs for Dnmt3a KO are denoted by with wavy lines

The CRISPR/Cas9 system was facilitated to generate Dnmt3a KO in CHO‐K1 cells. Basing on the coding conservation among different transcripts, we designed 2 pairs of single‐guide RNAs (sgRNAs), which targeted the conserved exon1 of the Dnmt3a transcript. Following the limiting dilution of genetically manipulated cells, PCR amplification was used to screen for monoclonal mutant cells. As shown in Figure 1A, 6 monoclones (3a‐30, 31, 32, 33, 40 and 41) harbouring indel mutations, which produce PCR product length polymorphisms, were isolated as Dnmt3a‐deficient candidate mutants and stored for further analyses.

**Figure 2:**
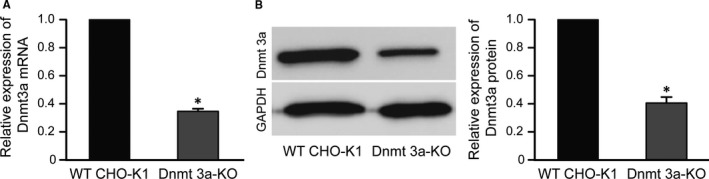
The expression levels of Dnmt3a in wild‐type (WT) and knockout (KO) CHO‐K1 cells. A, Expression of mRNA levels of Dnmt3a. Y‐exe values represent relative levels represent relative levels of mRNA obtained by the 2^ΔΔCt^ method. B, Western blot analysis. The optical density of each sample was measured and normalized using a GAPDH run on the same gel. The data are expressed as relative expression (ratio Dnmt3a/GAPDH). * indicates significant difference (*P* < .05) vs WT CHO‐K1 cells

PCR productions from 2 monoclones (3a‐30 and 40) were sequenced to validate the gene KO. The sequencing results revealed that the frame shift mutation occurred in the target region of the Dnmt3a gene (Figure 1B). The expression levels of Dnmt3 mRNA and protein were significantly decreased in the Dnmt3a‐deficient CHO‐K1 cells compared with the levels in the control CHO‐K1 cells (Figure 2, *P* < .05). These results indicated that Dnmt 3a gene was knocked out in CHO‐K1 cells.

### 3.2 Analysis of cells characteristics

**Figure 3:**
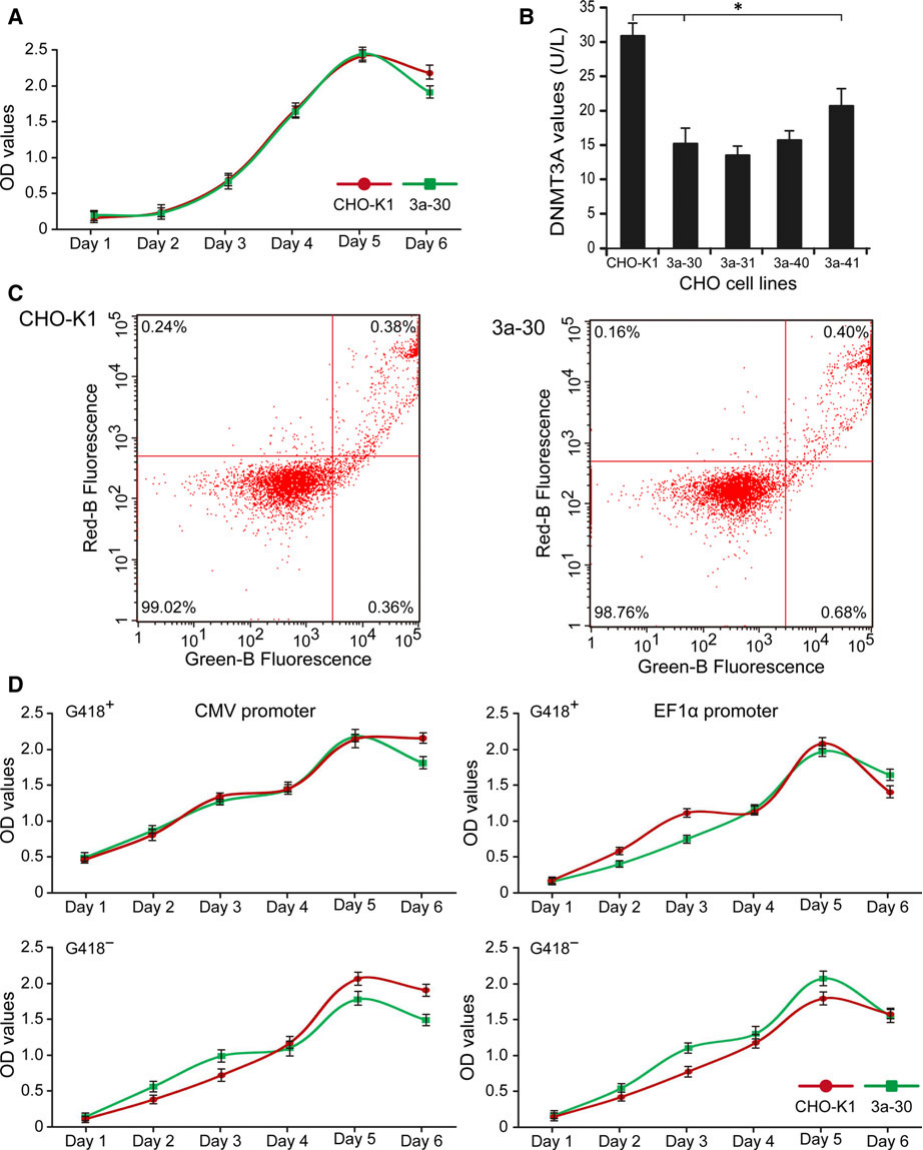
Detection of cell proliferation (A) and apoptosis (C) of Dnmt3a‐deficient and normal control CHO‐K1 cells. B, Analysis of DNMT3A by ELISA in the Dnmt3a‐deficient cell lines and normal control CHO‐K1 cells. D, Detection of cell proliferation in the stably transfected CHO cells. * indicates significant difference (*P* < .05) vs. CHO‐K1 cells

The detection of cell proliferation and apoptosis indicated that Dnmt3a KO did not alter the cell morphology and the growth status (Figure 3A,C). Growth characteristics of the Dnamt3a‐deficient cells, the CHO‐K1 cells and the cells stably transfected with CMV or EF1α were evaluated, as shown in Table 2. Results demonstrated that Dnmt3a deletion did not significantly affect the doubling times of the original CHO‐K1 cells and stably transfected cells. ELISA results showed that protein level was significantly decreased in the mutant cells (Figure 3B). Basing on the identification results, we selected one Dnmt3a‐deficient cell line (3a‐30) that had undergone dual allelic inactivation for further functional studies.

**Table 2:** Doubling times (*t*_D_) of the original Dnmt3a‐deficient and CHO‐K1 cells and of the stably transfected cells with the CMV or EF‐1α promoters

Cells	*t*_D_ (h)
CHO‐K1	17.15 ± 6.91
Dnmt3a deficient	14.42 ± 3.27

### 3.3 Transient expression and transfection efficiency

**Figure 4:**
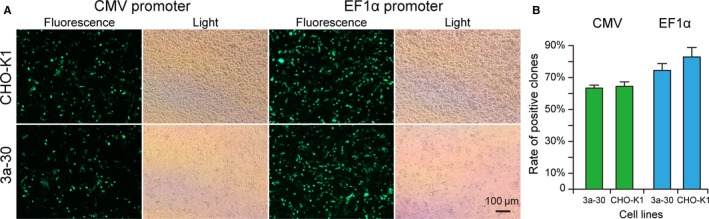
Transfection efficiency of the recombinant gene (A) and rates of positive clones (B) in the transiently transfected cell lines

Expression vectors driven by CMV or EF1α were transfected into 2 CHO cell lines, namely 3a‐30 and normal CHO‐K1 cells. After being transfected for 48 hours, the transfected cells were collected to observe the transfection efficiency under a fluorescence microscope. As shown in Figure 4A, no obvious differences were observed in the transfection efficiency of all of the transfected cells. Rates of positive clones in the cells transfected with CMV were slightly higher than those in the cells transfected with EF1α (Figure 4B). These results indicated that the Dnmt3a KO in CHO‐K1 cells had no effect on transient expression and transfection efficiency.

### 3.4 Effects of Dnmt3a KO on the recombinant expression in the stably transfected cells

**Figure 5:**
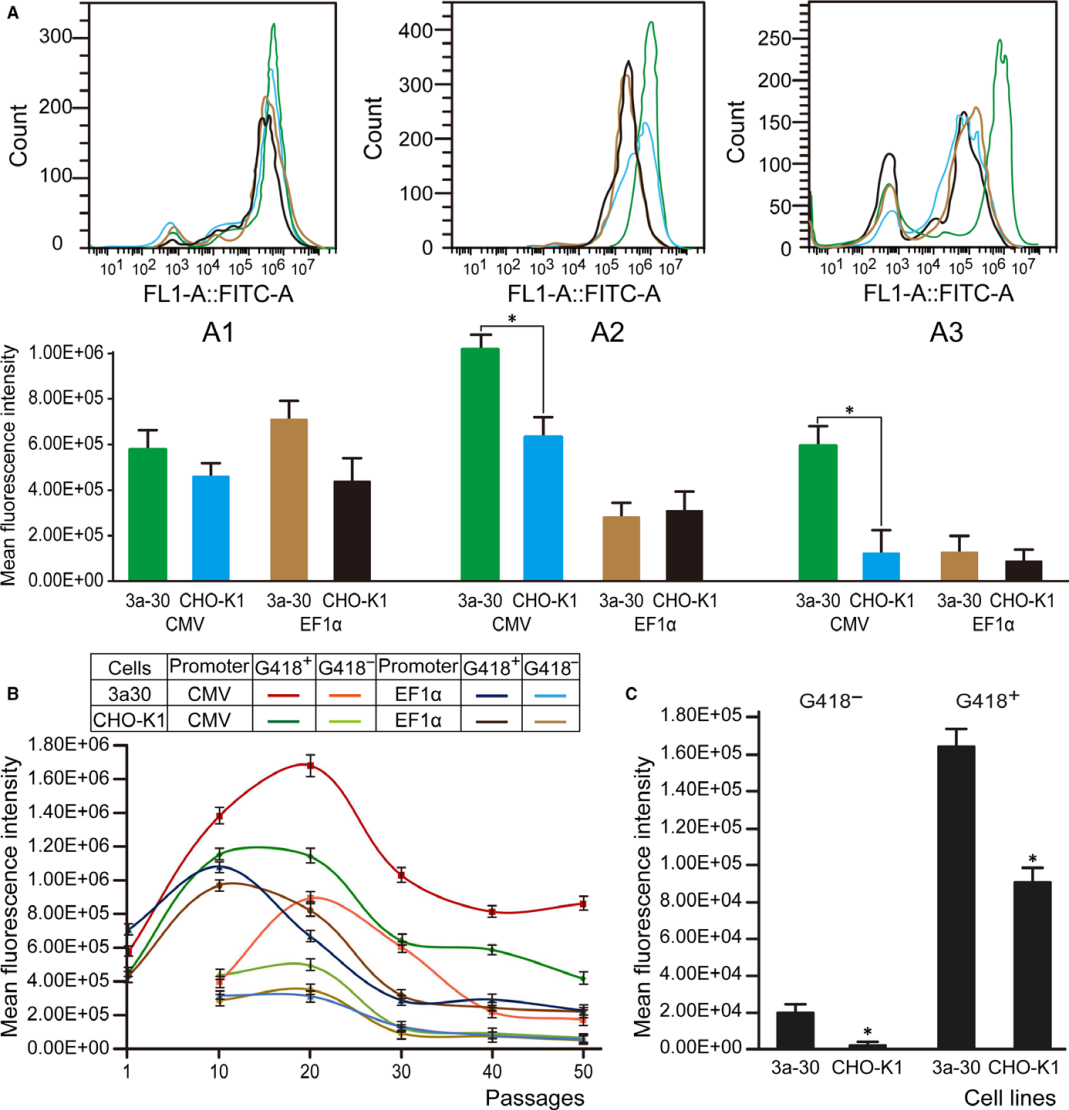
Stability of eGFP gene expression in the transfected cell pools of CHO‐K1 cells. A, MFIs of the eGFP protein levels determined by flow cytometry in the stably transfected cells (A1) and long‐term cultured cells for 30 passages under selection pressure with (A2) or without G418 (A3). B, MFIs of the eGFP protein levels in the Dnmt3a‐deficient 3a‐30 and control CHO‐K1 cells stably transfected with CMV or EF1α for 50 passages under selection pressure with or without G418. C, MFIs of the eGFP protein levels in the Dnmt3a‐deficient 3a‐30 and control CHO‐K1 cells stably transfected with CMV for 60 passages under selection pressure with or without G418. * indicates significant difference (*P* < .05) vs. CHO‐K1 cells

Four stable polycolonies from the 3a‐30 and normal control CHO‐K1 cells transfected with CMV or EF1α were obtained by screening with G418 for 14 days after transfection. These stably transfected cells exhibited similar cellular growth and proliferation behaviour (Figure 3D). As an indicator of the eGFP protein levels in the cells, the MFIs were determined through flow cytometry (Figure 5A). When the eGFP expression level in the control CHO‐K1 cells was 1.0, the relative eGFP expression levels in the 3a‐30 cells stably transfected with CMV and EF1α were 1.27 and 1.63, respectively, suggesting that the Dnmt3a‐deficient cells transfected with CMV or EF1α had higher expression levels than those of the control normal CHO‐K1 cells.

### 3.5 Significant improvements by Dnmt3a KO in long‐term expression stability

To verify the effects of Dnamt3a KO on the stability of transgene expression, polycolonies of the 3a‐30 and control CHO‐K1 cells stably transfected with CMV or EF1α were passaged under selection pressure in the presence (G418^+^) or absence (G418^−^) of G418 for 60 passages. The MFIs were detected to evaluate the intensity values of the expressed eGFP at 10, 20, 30, 40, 50 and 60 passages. The Dnmt3a‐deficient 3a‐30 cell line transfected with CMV exhibited the most stable and the highest expression levels regardless of the presence or absence of G418 (Figure 5). The transgene expression levels in the 3a‐30 and normal CHO cells stably transfected with EF1α exhibited loss of productivity during long‐term culture. Our results indicated that the Dnmt3a KO in CHO cells can enhance the long‐term stability of recombinant protein expression by using CMV for at least 50 passages (Figure 5B).

### 3.6 DNA methylation

To determine whether DNA methylation affected the long‐term transgene expression in CHO cell lines, we detected the DNA methylation of the global genomic DNA and the CMV or EF1α contained in the stably transfected CHO cells. The global DNA methylation percentage was (0.84 ± 0.08)% in untransfected 3a‐30 and (3.05 ± 0.13)% in CHO‐K1 cells. For CMV, the percentages of the DNA methylation of the global genomic DNA were (0.79 ± 0.11)% and (3.14 ± 0.17)% in the stably transfected 3a‐30 cells and normal CHO‐K1 cells, respectively, suggesting that the rate of global DNA methylation was significantly decreased in the Dnmt3a‐deficient cells (*P* < .05). For EF1α, the percentages of the DNA methylation of the global genomic DNA were (2.24 ± 0.21)% and (1.74 ± 0.19)% in the stably transfected 3a‐30 cells and normal control CHO‐K1 cells, respectively.

**Figure 6:**
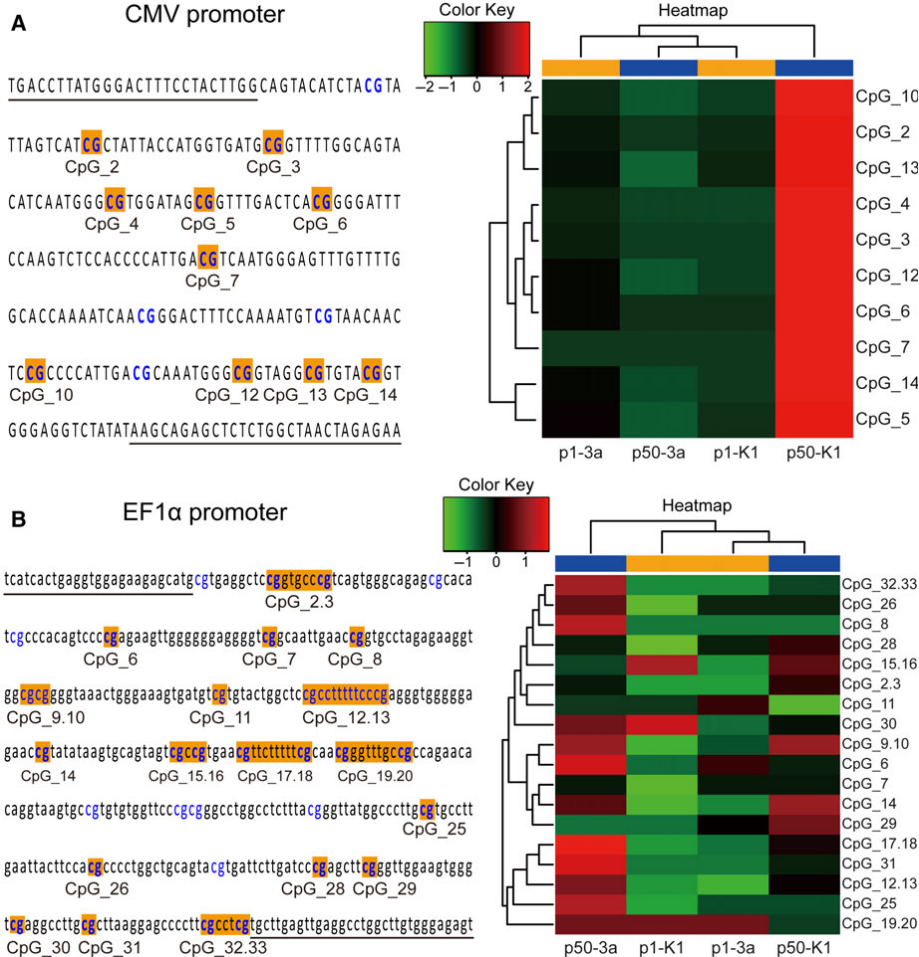
CpG sites in the amplicon and the hierarchical cluster analysis of methylation patterns of the CMV (A) or EF1α (B) regions in the stably transfected Dnmt3a‐deficient 3a‐30 (3a) and control CHO‐K1 (K1) cells at the start (p1) and 50 passages (p50)

We designed PCR primers to analyse via bisulphite sequencing a 284 bp fragment encompassing 14 CpG sites on the CpG island of CMV or a 459 bp fragment encompassing 33 CpG sites on the CpG island of EF1α in the stably transfected cell lines at 50 passages. Specifically, to perform DNA methylation analysis in the promoter region, we treated with bisulphite the total genomic DNA isolated from the stably transfected cell lines at 1 and 50 passages to convert the unmethylated cytosines into uracil. Methylated cytosines were left unchanged. After PCR amplification, the methylation state of the PCR product from the bisulphite‐modified DNA was determined using MALDI‐TOF Mass Array. The results revealed that the rate of DNA methylation in the analysed 10 CpG sites of CMV at high passage (P50) was high in the natural CHO cell lines but low at the start and at 50 passages in the 3a‐30 cell lines, thus verifying the high stability of transgene expression during long‐term culture (Figure 6A). By contrast, Dnmt3a KO exerted no influence on the reduction in the DNA methylation of EF1α in the transfected CHO cells. Rates of DNA methylation in the analysed 25 CpG sites of EF1α at the start and at high passage (P50) were low and had no differences in the transfected 3a‐30 and normal CHO cell lines (Figure 6B).

## 4 DISCUSSION

Previous reports have shown that the DNA hypermethylation in the promoter region can cause instability in stable recombinant CHO cell lines.^16^, ^17^ Previous report showed that Dnmt3a knockout CHO cell lines were constructed using the CRISPR‐Cas9 system without homologous regions.^28^ Thus, we established a DNA methyltransferase Dnmt3a‐deficient CHO cell line through CRISPR/Cas9 gene editing technology and investigated its effects on the long‐term productivity of permanently transfected CHO cells. We observed that Dnmt3a KO positively affected the maintenance of recombinant protein production in stably transfected CHO cells. In addition, the stability of transgene expression during long‐term cultivation could be distinctly enhanced in Dnmt3a‐deficient CHO cells transfected with CMV, presumably because of the distinct reduction in the DNA methylation of CMV in stably transfected Dnmt3a‐deficient CHO cells.

The CRISPR/Cas9 genome editing technology has been extensively applied for modifying a single gene into a genome‐wide regulation of genes through the rapid, easy and efficient engineering of mammalian genomes.^29^ This editing system is a highly efficient tool for generating gene disruptions in CHO cells 30 and has become highly important in CHO cell engineering as a means for expanding the product diversity and for controlling and improving the product quality and yields of recombinant protein therapeutics.^31^, ^32^, ^33^ Here, we demonstrated the efficacy of the CRISPR/Cas9 technology in CHO cells by generating site‐specific gene disruptions in Dnmt3a, which encodes the proteins involved in DNA methylation. To our knowledge, the gene KO of DNA methyltransferase has not been reported. To ensure effective outcome, we designed 2 pairs of single‐guide RNAs (sgRNAs), which targeted the conserved exon1 of the Dnmt3a transcript. The 2 sgRNAs yielded an indel frequency of up to 46.2% in Dnmt3a. Following the limiting dilution of genetically manipulated cells and screening by PCR amplification, 6 mutant monoclones harbouring indel mutations, which cause PCR product length polymorphisms, were isolated as Dnmt3a‐deficient candidates. Dnmt3a KO was confirmed in the mutant CHO cells by sequencing. Deep sequencing of the Dnmt3a KO revealed that the deletion of 199 bp nucleotides created by Cas9 resulted in frameshift mutations at the target sites. Moreover, the results of PCR amplification indicated that Dnmt3a KO was present in the Dnmt3a‐deficient CHO cells stably transfected with CMV or EF1α at 60 passages. This approach provides a fast and efficient strategy for generating gene KO to accelerate genome editing and synthetic biological efforts in CHO cells.

Cellular growth characteristics of Dnmt3a‐deficient and normal CHO‐K1 cell lines before and after transfection with CMV or EF1α were evaluated. For this purpose, the cell proliferation, apoptosis and doubling times were determined for the original Dnmt3a‐deficient, normal CHO‐K1 cells and stably transfected cells. In this work, the deletion of the Dnmt3a gene did not significantly affect the doubling times of the mutant CHOs and transfected cells. Characteristics of cell proliferation and apoptosis showed no differences in the cell morphology and growth status of the mutant and normal CHO cells. Challen et al^34^ reported that mice with Dnmt3a KO in the bone marrow produced phenotypically normal hematopoietic stem cells. All of the Dnmt3a KO clones grew well and were morphologically normal, indicating that the deletion of the Dnmt3a gene had no obvious effects on the morphology and viability of CHO cells. This finding was consistent with that of previous studies, wherein the knockouts in human and mouse ESCs were dependent on DNA methylation levels than on enzymes in the teratoma assay.^35^, ^36^

The hCMV‐MIE provides high levels of transgene expression; however, its loss of productivity has been reported with prolonged cultivation time.^37^, ^38^ hCMV‐MIE is prone to transgene silencing, which is associated with DNA methylation.^16^, ^17^, ^37^, ^39^ Moreover, recombinant CHO cell lines with EF1α exhibited loss of productivity during long‐term culture.^26^, ^40^ Therefore, to monitor the effects of Dnmt3a‐deficient CHO cells on transgene expression, we used 2 promoters, namely CMV and EF1α, to drive the recombinant gene expression. For this purpose, we chose an eGFP reporter, which enables cytometric analysis of the reporter gene expression. The eGFP reporter plasmids driven by CMV or EF1α were transfected into Dnmt3a‐deficient 3a‐30 and normal control CHO cells, and 4 independent pools of permanently transfected cells were selected with G418. These cells were cultivated for over 5 months (50 passages) in the presence or absence of the selection agent G418. The MFIs of the eGFP expression for each cell pool were measured by cytometry at 0, 10, 20, 30, 40 and 50 passages. All of the stably transfected cells in the presence of G418 showed elevated expression levels at early passages (up to P20); however, the eGFP expression was strongly increased in the 3a‐30 cells transfected with CMV, which displayed distinctively higher eGFP values than those of the other stably transfected CHO cells. After cultivation for 50 passages, the 3a‐30 cells transfected with CMV still displayed high levels of eGFP, whereas the eGFP expression of the other transfected cells had obviously dropped, particularly in the cells transfected with EF1α. In the absence of G418, the eGFP expression in the CHO cells transfected with CMV or EF1α was markedly decreased throughout the long‐term cultivation, except for the 3a‐30 cells transfected with CMV, which sustained high levels of stable eGFP expression for over 30 passages. Overall, these results suggested that Dnmt3a KO can significantly delay the loss of CMV‐driven recombinant gene expression.

To investigate whether Dnmt3a KO could affect the methylation levels at CpG sites within the promoter region, we measured the methylation levels of CpG sites in the CMV and EF1α regions through bisulphite conversion coupled with PCR amplification and MALDI‐TOF Mass Array. We observed a distinct methylation pattern at 50 passages in the CMV promoter region of the normal transfected control CHO cells, which showed the highest rate of DNA methylation. In particular, we detected low DNA methylation in the promoter region at start and at 50 passages in the transfected 3a‐30 cells, which showed high stability of transgene expression during long‐term culture. Moritz et al^19^ reported that the cytosine located at 41 and 179 bp upstream of the transcription start site (C‐41G and C‐179G) is among the most frequently methylated sites in CMV and that a single mutation of C‐179 into G can significantly stabilize the production of recombinant proteins under the control of CMV in permanently transfected CHO cells. The rate of DNA methylation at these 2 CpG sites (ie CpG_14 and CpG_5) detected in this study was the highest (0.75 and 0.54, respectively) in the transfected normal CHO cells at 50 passages and was significantly lower (0.08 and 0.11, respectively) in the transfected 3a‐30 cells. These results indicated that theses‐specific sites of DNA methylation in the promoter sequences, such as CMV, are critical sites that prevent or inhibit transcription.^41^ Compared with CMV, EF1α displayed decreased rates of DNA methylation, which presented no significant differences between the 3a‐30 and normal control CHO cells at the start and at 50 passages, suggesting that the density of methylation in a given region determines whether or not transcription would be prevented.^42^ The possible reason is that the effect of CpG methylation on gene expression is context dependent^43^; that is, the degree of CpG methylation change, the density or the genomic location of CpG, methyl‐CpG “readers” and TF binding are possible factors that affect the ultimate effect of DNA methylation on gene expression. Furthermore, Veith et al^13^ revealed low methylation in 18 CpG sites of the EEF1Α1 promoter region in 4 PT1‐CHO cell lines with a loss of productivity at high passage (P50). These findings implied the distinct improvement in the long‐term transgene stability by Dnmt3a deletion due to the reduction in DNA methylation in the CMV promoter region. Aside from epigenetic promoter silencing, the loss of transgene expression in the transfected cells could primarily reflect the integration of genomic DNA and the loss of recombinant mRNA expression.^8^ Moreover, we detected the copy number and mRNA expression of the eGFP gene in each cell line by performing qPCR. We found no clear correlation between the eGFP gene copy numbers or mRNA levels and the eGFP expression levels for both CMV and EF1α in the stably transfected cell lines (data not shown).

In summary, Dnmt3a‐dificent CHO cells are a potential alternative expression system to traditional methods. This approach offers a new platform for improving the transgene expression stability in recombinant CHO cells. Obviously, further studies on Dnmt3a‐deficient CHO cells and DNA methylation may help elucidate the underlying molecular mechanisms. Our results may be promoter‐specific and follow‐up studies that consider more promoters could provide additional insight into the effects of Dnmt3a KO on the long‐term expression stability for the large‐scale production of recombinant expression in CHO and other mammalian cells. Therefore, we believe that the development of the Dnmt3a‐deficient CHO cell line will be greatly beneficial to a broad community of users.

## CONFLICT OF INTERESTS

The authors declare that they have no competing interests.

## AUTHOR CONTRIBUTIONS

W.T.Y. and J.Y.L. conceived and designed the study and analysed the results. G.X., L.J.T. and Q.L.L. performed the experiments. G.X., J.Y.L. and W.T.Y. and analysed the data. J.Y.L. and W.T.Y. wrote the paper. All authors read, edited and approved the final manuscript.
